# Nevirapine Resistance and Breast-Milk HIV Transmission: Effects of Single and Extended-Dose Nevirapine Prophylaxis in Subtype C HIV-Infected Infants

**DOI:** 10.1371/journal.pone.0004096

**Published:** 2009-01-01

**Authors:** Anitha Moorthy, Amita Gupta, Ramesh Bhosale, Srikanth Tripathy, Jayagowri Sastry, Smita Kulkarni, Madhuri Thakar, Renu Bharadwaj, Anju Kagal, Arvind V. Bhore, Sandesh Patil, Vandana Kulkarni, Varadharajan Venkataramani, Usha Balasubramaniam, Nishi Suryavanshi, Carrie Ziemniak, Nikhil Gupte, Robert Bollinger, Deborah Persaud, for the India SWEN Study Team

**Affiliations:** 1 Department of Molecular Microbiology and Immunology, Johns Hopkins Bloomberg School of Public Health, Baltimore, Maryland, United States of America; 2 Department of Adult Infectious Disease, Johns Hopkins University School of Medicine, Baltimore, Maryland, United States of America; 3 Byramjee Jeejeebhoy Medical College, Pune, India; 4 National AIDS Research Institute, Pune, Maharashtra, India; 5 India SWEN Study Team, Pune, Maharashtra, India; 6 Department of Pediatrics, Johns Hopkins University School of Medicine, Baltimore, Maryland, United States of America; National Institute of Child Health and Human Development, United States of America

## Abstract

**Background:**

Daily nevirapine (NVP) prophylaxis to HIV-exposed infants significantly reduces breast-milk HIV transmission. We assessed NVP-resistance in Indian infants enrolled in the “six-week extended-dose nevirapine” (SWEN) trial who received single-dose NVP (SD-NVP) or SWEN for prevention of breast-milk HIV transmission but who also acquired subtype C HIV infection during the first year of life.

**Methods/Findings:**

Standard population sequencing and cloning for viral subpopulations present at ≥5% frequency were used to determine HIV genotypes from 94% of the 79 infected Indian infants studied. Timing of infection was defined based on when an infant's blood sample first tested positive for HIV DNA. SWEN-exposed infants diagnosed with HIV by six weeks of age had a significantly higher prevalence of NVP-resistance than those who received SD-NVP, by both standard population sequencing (92% of 12 vs. 38% of 29; p = 0.002) and low frequency clonal analysis (92% of 12 vs. 59% of 29; p = 0.06). Likelihood of infection with NVP-resistant HIV through breast-milk among infants infected after age six weeks was substantial, but prevalence of NVP-resistance did not differ among SWEN or SD-NVP exposed infants by standard population sequencing (15% of 13 vs. 15% of 20; p = 1.00) and clonal analysis (31% of 13 vs. 40% of 20; p = 0.72). Types of NVP-resistance mutations and patterns of persistence at one year of age were similar between the two groups. NVP-resistance mutations did differ by timing of HIV infection; the Y181C variant was predominant among infants diagnosed in the first six weeks of life, compared to Y188C/H during late breast-milk transmission.

**Conclusions/Significance:**

Use of SWEN to prevent breast-milk HIV transmission carries a high likelihood of resistance if infection occurs in the first six weeks of life. Moreover, there was a continued risk of transmission of NVP-resistant HIV through breastfeeding during the first year of life, but did not differ between SD-NVP and SWEN groups. As with SD-NVP, the value of preventing HIV infection in a large number of infants should be considered alongside the high risk of resistance associated with extended NVP prophylaxis.

**Trial Registration:**

ClinicalTrials.gov NCT00061321

## Introduction

In 2007, an estimated 420,000 infants worldwide were infected with human immunodeficiency virus (HIV) through *in utero*, peripartum, or breast-milk transmission [1]. Antiretroviral drugs and avoidance of breastfeeding can reduce the risk of mother-to-child transmission (MTCT) of HIV to less than 2% [2], [3] from 25–35% [4]. Single-dose nevirapine (SD-NVP), used to help prevent perinatal HIV infection in resource-constrained settings, reduces transmission by 50% but has limited effect on subsequent breast-milk transmission [5].

The overall risk of HIV transmission through breastfeeding is estimated to be 10%, with the greatest risk by the first six to 14 weeks of life, a period in which 60–70% of breast-milk transmission occurs [6]. Despite this risk, the World Health Organization (WHO) recommends that HIV-infected mothers exclusively breastfeed for at least six months to improve infant survival [7], [8]. To maintain breastfeeding and reduce transmission risk, nevirapine (NVP) dosing could be extended into the period of greatest risk. Two recently completed randomized controlled trials showed that daily dosing of NVP for up to six weeks of age (“six week extended-dose NVP” [SWEN] trial) or NVP with or without zidovudine (AZT) administered to 14 weeks of age (PEPI-Malawi trial) reduced breast-milk transmission by 46% and 66%, respectively [9], [10].

It is known, however, that SD-NVP prophylaxis comes with the risk of drug resistance if prevention fails. Pooled estimates showed that 36% (19–76%) of women and 53% (36–87%) of infants have detectable NVP resistance (NVP-R) mutations 6–8 weeks after exposure to SD-NVP [11]. Importantly, these mutations decrease in frequency by 12–18 months following exposure to SD-NVP, and can no longer be detected by standard population sequencing [12], [13], [14], and detection requires more sensitive assays [15], [16], [17]. The selection of NVP-R after SD-NVP may reduce the likelihood of achieving virologic suppression with NVP-containing highly active antiretroviral therapy (HAART) regimens, although the impact on treatment outcomes remains unclear [18], [19].

Ugandan infants receiving extended daily NVP prophylaxis and diagnosed with subtypes A or D infection the first six weeks of life have been observed to have a higher risk of drug resistance than infants receiving SD-NVP [20]. However, the risk of NVP-R arising as a result of unsuccessful daily NVP prophylaxis in subtype C infected infants is not known, nor is the continuing risk from breast-milk transmission after prophylaxis during the first year of life. And, while it is generally true that SD-NVP exposure results in a lower risk of drug resistance in HIV subtype A or D infected individuals than for those infected with subtype C [21], the relative effect of extended NVP prophylaxis on resistance among HIV subtypes is not known. To address these questions, we compared the prevalence of NVP-R mutations following administration of single- or extended-dose NVP to subtype C infected Indian infants enrolled in the SWEN trial who became infected despite prophylaxis, and in whom the timing and mode of HIV infection were determined.

## Methods

### Study Participants and Intervention

The SWEN trial was conducted in Ethiopia, India, and Uganda from February 2001 to March 2007 [10]. Informed consent was obtained as part of the parent trial as described previously [10]. Briefly, written informed consent was obtained where possible; and verbal consent was obtained when subjects were unable to read and subsequently documented in writing by a witness. HIV drug resistance testing was performed on de-identified Indian plasma samples shipped to Johns Hopkins University for analysis and approved by the Johns Hopkins Institutional Review Board. In this trial, HIV-infected pregnant women cared for at Byramjee Jeejeebhoy Medical College in Pune, India, were enrolled during their antenatal visit, at delivery, or within one week post-partum. Women enrolled before delivery were offered short-course AZT [22] in addition to SD-NVP for prevention of MTCT of HIV, but acceptance was the individual's choice [10]. Infants were randomized at birth to receive SD-NVP plus multivitamins, or 5 mg of NVP once daily with multivitamins from one week to six weeks of age [10]. To limit exposure of an HIV-infected infant to monotherapy with NVP, study drugs were discontinued once HIV infection was detected. HIV-infected infants were treated according to local clinical care guidelines [23]. Overall, 34% of 730 women did not receive intrapartum SD-NVP, primarily because they were unaware of their HIV status at the time of delivery and were enrolled post-partum.

### Estimation of the Timing and Mode of Infection

Blood samples were collected to determine HIV infection status in infants at 48 hours, weeks 1, 2, 4, 6, 10, 14 and months 6, 9 and 12 through detection of HIV DNA by polymerase chain reaction (PCR) using a validated in-house assay [24]. HIV quantitative RNA testing (Roche Amplicor HIV Monitor test version 1.5, F. Hoffmann-La Roche Ltd, Basel, Switzerland) was used to confirm infection at the next scheduled study visit. Based on the time of first positive HIV DNA PCR, infants were assigned to one of the following categories reflecting the mode of infection: *in utero* (positive by 48 hours); peripartum/early-breastfeeding (positive from weeks one through six); late-breastfeeding (positive after six weeks).

### Genotyping

Infants were only included in the analysis of NVP-R mutations if plasma samples for genotyping were available at the first positive HIV DNA PCR test (n = 13) or the first (n = 43) or second (n = 18) subsequent visits. Unlike other studies of SD-NVP prophylaxis, analysis of NVP-R at a fixed age after NVP exposure could not be performed in the Indian cohort because plasma samples were collected sequentially only until the diagnosis of HIV was confirmed, after which plasma samples were not collected at regular intervals. The median age at genotyping for infants diagnosed with HIV in the first six weeks of life for the cohort was 29 days (interquartile range [IQR]: 22–43 days). Seven SD-NVP exposed women who transmitted HIV during late-breastfeeding and who had plasma samples available at six months after delivery were also genotyped. HIV genotyping was performed by standard population sequencing, which detects mutant viral subpopulations present at >20% frequency [25], [26] using a previously published assay [27]. Sequences were assembled in Bioedit Version 7, and mutation errors from PCR amplification were removed [28]. Phylogenetic trees were constructed by neighbor-joining method (1000 bootstrap permutations) using Mega Version 4.0 [29] with HIV subtype B and C reference sequences from the Los Alamos HIV database [30] to determine patient specificity and genetic relatedness between infant and maternal sequences. NVP-R was defined by mutations present at the following amino acid sites: L100I, K101E/P, K103N/S, V106A/M, V108I, Y181C/I/V, Y188C/L/H, or G190A/S/E based on recommendations from International AIDS Society-USA Drug Resistance Mutations and Stanford University drug-resistance database [31], [32]. Nucleotide sequences have been deposited in GenBank, and the accession numbers are FJ520232 to FJ524294.

### Low Frequency Variant Analysis

For infants diagnosed with HIV infection within the first 14 weeks of life and in whom NVP-R mutations were not detected by standard population sequencing, extensive cloning was used to detect mutant viral subpopulations present at ≥5% [14]. PCR-amplified products were cloned into TOPO cloning vectors (Invitrogen, Carlsbad, CA), and up to 96 clones per subject were selected for sequencing on an ABI 3730 sequencer (Agencourt Bioscience Corporation, Beverly, MA). Low frequency variant analysis was also performed on: infants infected after 14 weeks of age who had wild-type HIV infection by standard population sequencing and whose mothers received SD-NVP, the seven mothers with available samples collected six months postpartum who transmitted HIV through late-breastfeeding, and also on 10 infants (5 in each intervention group) who had NVP-R detectable by standard population sequencing at first analysis and had follow-up samples for assessing persistence of NVP-R.

### Statistical Analysis

The outcome reported here, as prespecified in the original trial protocol, was to compare NVP-R by timing and mode of HIV infection among infants who received SD-NVP or SWEN for prevention of breast-milk HIV transmission. However, an “as-treated” analysis instead of an intent-to-treat analysis was performed to evaluate NVP exposure on resistance outcomes in infants. Additional outcomes assessed were types of NVP-R mutations, patterns and prevalence of persistence of NVP-R mutations, and genotypes from seven SD-NVP exposed women six months postpartum whose infants became infected during late breastfeeding. Characteristics of mothers and infants in each intervention group were compared using Fisher's exact test for categorical variables and Mann-Whitney test for continuous variables. Data analyses were performed using STATA software, version 10.0 (StataCorp LP, College Station, TX).

## Results

NVP-R analysis was performed on 74 of 96 (77%) HIV subtype C infected Indian infants in the SWEN trial. Samples were missing from 17 infants (18%) who were either lost to follow-up (n = 4), died (n = 1), or had insufficient or no available plasma sample within two visits of diagnosis of HIV infection (n = 12). HIV-1 genotyping was successful in 74 of 79 (94%) evaluable infants. Of these 74 infants, 55% were infected *in utero* (n = 22) or through peripartum/early-breastfeeding transmission (n = 19), and 45% were infected through late-breastfeeding (n = 33). While analysis of NVP-R was not performed at a fixed age for the cohort, the median age at genotyping was similar to other published studies of NVP-R following SD-NVP exposure [11]. For infants infected *in utero* and through peripartum/early-breastfeeding, the median age at genotyping was 22 days (IQR: 17–28 days) and 43 days (IQR: 30–71 days), respectively. For those infected through late-breastfeeding, the median age at genotyping was 189 days (IQR: 182–368 days) with a median interval between HIV diagnosis and genotyping of 39 days (IQR: 28–89 days). Infants randomized to SWEN received study drugs for a median of 14 days (IQR: 9–15 days) in the *in utero* group; 29 days (IQR: 22–35 days) in peripartum/early-breastfeeding group; and 35 days (IQR: 33–35 days) in late-breastfeeding group. Two SWEN-exposed infants diagnosed with infection in the first six weeks of life were still receiving NVP when genotyping was performed. Phylogenetic analyses confirmed that infant sequences were patient-specific and co-mingled with HIV subtype C Indian reference sequence and corresponding maternal sequences (data not shown).

Drug resistance was therefore assessed in 25 infants who received SWEN and in 49 infants who received SD-NVP. A higher proportion of mothers of infants who received SWEN were started on combination antiretroviral therapy prior to or following delivery than mothers of infants who received SD-NVP (32% vs. 8%; p = 0.02) (Table 1). Most women (11 of 12) in both groups were treated after delivery; however, genotypes in 10 of the 11 corresponding infants were obtained prior to initiation of maternal antiretroviral therapy and did not likely influence the resistance outcomes in these infants.

**Table 1:** Infant and maternal characteristics by infant intervention.

Infant and Maternal Characteristics	SWEN (n = 25)	SD-NVP (n = 49)	p-value*
Male	13 (52)	24 (49)	0.81
Median Birth Weight (grams [IQR])	2600 (2500, 3000)	2500 (2250, 2950)	0.36
Preterm Birth	2 (8)	11 (22)	0.20
Median duration of breastfeeding (days [IQR])	122 (98, 188)	182 (98, 337)	0.38
Infant median CD4% at time of confirmed diagnosis (IQR)	30 (22, 38)	29 (20, 36)	0.79
Infant median log_10_ viral load at time of confirmed diagnosis (copies/ml [IQR])	5.4 (5.3, 5.8)	5.6 (5.2, 5.8)	0.82
Maternal Age (years [IQR])	25 (23, 26)	22 (21, 26)	0.35
Maternal Education≥secondary level	19 (76)	30 (61)	0.30
Maternal median CD4 counts at time of delivery (cells/mm^3^ [IQR])	316 (238, 454)	320 (192, 522)	0.47
Maternal median log_10_ viral load at time of delivery (copies/ml [IQR])	4.9 (4.2, 5.2)	4.5 (3.8, 5.1)	0.24
Maternal intrapartum SD-NVP	12 (48)	30 (61)	0.33
Maternal antepartum AZT	4 (16)	14 (29)	0. 27
Maternal antiretroviral therapy †	8 (32)	4 (8)	0.02

Data are number of patients (%) unless stated otherwise.

SWEN = up to six weeks of extended-dose nevirapine; SD-NVP = single-dose nevirapine; AZT = zidovudine; IQR = interquartile range.

* All p-values generated using Fisher's exact test for categorical variables and Mann-Whitney test for continuous variables.

† Maternal antiretroviral therapy was started after delivery in 11 of 12 women in both groups. Genotypes in 10 of these 11 corresponding infants were obtained prior to initiation of maternal antiretroviral therapy.

**Figure 1:**
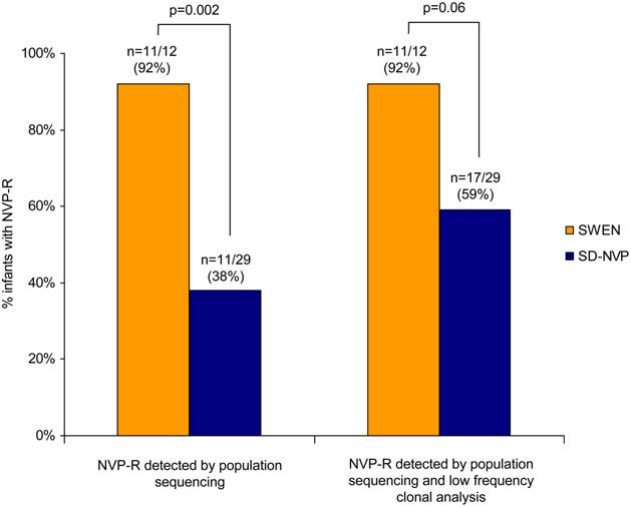
SWEN and SD-NVP exposed HIV-infected infants diagnosed within the first six weeks of life with NVP-R. SWEN = up to six weeks of extended-dose nevirapine; SD-NVP = single-dose nevirapine; NVP-R = nevirapine resistance. P-values generated using Fisher's exact test.

In an “as-treated” analysis, we found that infants diagnosed with HIV infection in the first six weeks of life who received SWEN were significantly more likely to have NVP-R mutations detected by standard population sequencing compared to infants who received SD-NVP (92% of 12 vs. 38% of 29; p = 0.002; Figure 1). Exclusion of the 22 infants infected *in utero* (who had established HIV infection at birth, could not have benefited from the intervention, and might be expected to have higher prevalence of drug resistance), showed that infants infected through peripartum/early-breastfeeding who received SWEN were also significantly more likely to have NVP-R mutations than infants who received SD-NVP (100% of 4 vs. 27% of 15; p = 0.008).

However, since standard population sequencing underestimates prevalence of NVP-R arising during prophylaxis, we identified through clonal analysis (median 85 clones/patient) an additional six of 15 SD-NVP-exposed infants diagnosed within the first six weeks of life with NVP-R in whom cloning was successful. Cloning was not successful in the one SWEN-exposed infant with wild-type infection by standard population sequencing. Prevalence of NVP-R in the SWEN group remained higher when low frequency variants were also considered (92% of 12 vs. 59% of 29; p = 0.06; Figure 1; Table 2). This trend was also evident when only infants infected during the first six weeks of life through peripartum/early-breastfeeding were included, but this was not statistically significant (100% of 4 vs. 53% of 15; p = 0.25; Table 2).

**Table 2:** NVP-R mutations among SWEN and SD-NVP exposed infants.

Infant Intervention	Timing and Mode of Infection	Genotyping Method	Types of NVP-R Mutations, n/n (%)							>1 NVP-R Mutation, n/n (%)	Mixture with WT/NVP-R, n/n (%)	Clonal Frequency, Median% (Range)
			K101E	K103N	V106A/M	V108I	Y181C	Y188C/H	G190A/E			
**SWEN**	***In utero***	**Population** *	-	2/13 (15)	1/13 (8)	1/13 (8)	6/13 (46)	1/13 (8)	2/13 (15)	5/7 (71)	5/7 (71)	n/a
	**Peripartum/early-breastfeeding**	**Population** *	-	1/4 (25)	1/4 (25)	-	1/4 (25)	1/4 (25)	-	0/4 (0)	0/4 (0)	n/a
	**Late-breastfeeding**	**Population**	-	1/2 (50)	-	-	-	1/2 (50)	-	0/2 (0)	0/2 (0)	n/a
		**Cloning**	-	1/2 (50)	-	-	-	1/2 (50)	-	0/2 (0)	n/a	1.3 (1.1–1.4)
**SD-NVP**	***In utero***	**Population**	1/10 (10)	2/10 (20)	1/10 (10)	-	5/10 (50)	-	1/10 (10)	2/7 (29)	4/7 (57)	n/a
		**Cloning**	-	1/5 (20)	1/5 (20)	1/5 (20)	-	1/5 (20)	1/5 (20)	1/2 (50)	n/a	5.4 (1.1–9.7)
	**Peripartum/early-breastfeeding**	**Population**	-	2/5 (40)	1/5 (20)	-	1/5 (20)	1/5 (20)	-	1/4 (25)	3/4 (75)	n/a
		**Cloning**	-	-	1/5 (20)	1/5 (20)	2/5 (40)	-	1/5 (20)	1/4 (25)	n/a	5.3 (1.1–16.5)
	**Late-breastfeeding**	**Population**	-	1/3 (33)	-	-	-	1/3 (33)	1/3 (33)	0/3 (0)	1/3 (33)	n/a
		**Cloning**	-	1/5 (20)	-	-	-	3/5 (60)	1/5 (20)	0/5 (0)	n/a	2.7 (1.2–9.5)

NVP-R = nevirapine resistance; WT = wild-type; SD-NVP = single-dose nevirapine; SWEN = up to six weeks of extended-dose of nevirapine; n/a = not applicable.

* Cloning was not successful in the one *in utero* infected infant with wild-type HIV in the SWEN group.

Among *in utero* infected infants, 80% of 10 infants randomized to SWEN received extended doses of NVP because of delays in diagnosis. The proportion of *in utero* infected infants who received SWEN or SD-NVP and who possessed NVP-R were 88% of 8 vs. 50% of 14, respectively (p = 0.17) using standard population sequencing, and 88% of 8 vs. 64% of 14, respectively (p = 0.35) by low frequency clonal analysis.

**Figure 2:**
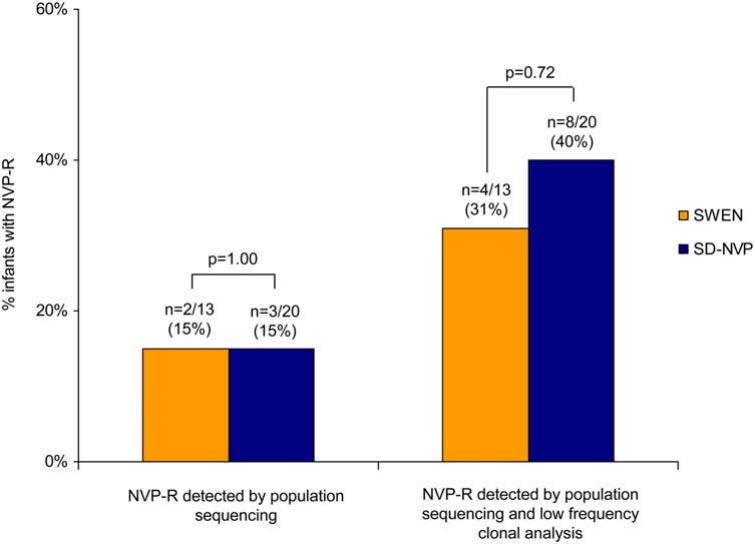
SWEN and SD-NVP exposed infants infected with HIV after six weeks of age through late-breastfeeding with NVP-R. SWEN = up to six weeks of extended-dose nevirapine; SD-NVP = single-dose nevirapine; NVP-R = nevirapine resistance. P-values generated using Fisher's exact test.

Because infants were serially tested for HIV infection throughout the first year of life, we were able to assess the likelihood of acquiring NVP-R if infected after six weeks of age through late breast-milk transmission, a rate which was similar for both intervention groups. Using standard population sequencing, 15% of 13 infants who received SWEN and 15% of 20 infants who received SD-NVP had drug resistance (p = 1.00; Figure 2). The proportion of infants infected through late-breastfeeding with NVP-R at low frequencies was substantial but similar for SWEN and SD-NVP groups when examined by clonal analysis (31% of 13 vs. 40% of 20; p = 0.72; Figure 2; Table 2).

The types and number of NVP-R mutations were similar across intervention groups, but differed by timing of infection (Table 2). Among infants diagnosed in the first six weeks of life, the predominant mutations detected by standard population sequencing in SWEN and SD-NVP groups were Y181C (41% of 17 vs. 32% of 25; p = 0.74) and K103N (18% of 17 vs. 20% of 25; p = 1.00), respectively (Table 2). NVP-R variants present as mixtures with wild-type HIV were similar in both intervention groups (Table 2). Seventy-one percent of infants infected *in utero* who received SWEN had more than one NVP-R mutation compared to 29% who received SD-NVP, but this difference was not statistically significant (p = 0.29; Table 2). However, NVP-R mutations in infants infected through late-breastfeeding differed from those diagnosed in the first six weeks of life. In the event of late-breastfeeding transmission, Y188C/H was the most common mutation present and was found at similar frequencies for infants in the SWEN and SD-NVP groups (50% of 4 vs. 50% of 8; p = 1.00; Table 2).

To further investigate breast-milk transmission of NVP-R HIV among infants infected after six weeks of age, we examined HIV genotypes six months after delivery in the seven available samples from women exposed to SD-NVP who also transmitted HIV through late-breastfeeding. All seven women and six of the seven paired infants had wild-type HIV detectable by standard population sequencing at the time of HIV diagnosis (Table 3). However, NVP-R was present at low frequencies (73 clones/patient) in four women (57%; median clonal frequency of 5.6%; range 2.4%–14.5%). K103N was the most common mutation in three of these four women (75%; Table 3). Although paired maternal and infant sequences were phylogenetically related (data not shown), mutations detected at low frequencies in infants infected through late-breastfeeding were different from those observed in their mothers (Table 3).

**Table 3:** Late breast-milk transmission of NVP-R HIV in seven mother-infant pairs.

Infant Intervention	Infant ID	Infant Age at HIV Diagnosis (Months)	Infant Genotype at Diagnosis (Population Sequencing)	Infant Genotype at Diagnosis (Clonal Analysis)	Maternal Genotype at 6 Months Postpartum (Population Sequencing)	Maternal Genotype at 6 Months Postpartum (Clonal Analysis)
SWEN	6	2.5	WT	Y188H	WT	K103N
	38	2.5	WT	WT	WT	K103N
SD-NVP	10	2.5	WT/G190A	n/a*	WT	K103N
	20	6	WT	WT	WT	Y181C, Y188C
	61	9	WT	WT	WT	WT
	40	12	WT	Y188H	WT	WT
	74	12	WT	WT	WT	WT

NVP-R = nevirapine resistance; WT = wild-type HIV; SWEN = up to six weeks of extended-dose of nevirapine; SD-NVP = single-dose nevirapine; n/a = not applicable.

* G190A detected as a mixture with wild-type HIV using standard population sequencing therefore, cloning was not performed in this patient.

Differences in persistence of NVP-R mutations were assessed in a small number of infants exposed to SWEN (n = 5) or SD-NVP (n = 5) who had available plasma samples and also had NVP-R mutations detected by standard population sequencing at the time of HIV diagnosis (Table 4). Loss of detectable NVP-R mutations using standard population sequencing was observed at a median of 12 months of age in infants who received SWEN (60% of 5) or SD-NVP (80% of 5) (Table 4). However, most infants with wild-type HIV had persistent NVP-R mutations at low frequencies (86%; median clonal frequency of 3.1%; range 1.2%–12.8%), with similar rates and mutation patterns for both intervention groups (Table 4).

**Table 4:** Extent of persistence of NVP-R mutations in SWEN and SD-NVP exposed infants.

Infant Intervention	Infant ID	Timing and Mode of Infection	Genotype at Diagnosis	Age at Follow-up (Months)	Genotype at Follow-up (Population Sequencing)	Genotype at Follow-up (Clonal Analysis)
**SWEN**	11	*In utero*	V106M, Y181C	12	WT	WT
	62	*In utero*	Y181C	7	Y181C	Y181C
	57	Peripartum/early-breastfeeding	K103N	7	K103N	K103N
	49	Peripartum/early-breastfeeding	Y188C	15	WT	Y188C
	56	Late-breastfeeding	Y188C	12	WT	V108I
**SD-NVP**	22	*In utero*	K103N, V106M	12	WT	V108I
	71	Peripartum/early-breastfeeding	K103N	13	WT	G190E
	13	Late-breastfeeding	K103N	6	WT	G190A
	10	Late-breastfeeding	G190A	12	K103N	K103N, V108I
	32	Late-breastfeeding	Y188C	12	WT	Y188C/H

NVP-R = nevirapine resistance; SWEN = up to six weeks of extended-dose nevirapine; SD-NVP = single-dose nevirapine.

## Discussion

Within the context of the SWEN trial to prevent breast-milk HIV transmission, we found that prevalence of NVP-R was higher for Indian infants who became subtype C HIV-infected in the first six weeks of life despite receiving daily NVP than for those who had received SD-NVP, a relationship that held even when low-frequency variants were considered. Furthermore, we found a substantial proportion of infants infected through breast-milk transmission during the first year of life had NVP-resistant HIV when low frequency variants were considered. The types of NVP-resistance mutations were similar between the two intervention groups but differed by timing of infection, where the Y181C variant was predominant in infections occurring in the first six weeks of life compared to the Y188C/H variant associated with late breast-milk infections. The pattern of persistence of NVP-R was similar at both population and low frequency levels at a median of one year of age, although only a small number of infants were studied.

The high prevalence of NVP-R in this study is not surprising given the long half-life of the drug [33] and the rapid selection of NVP-R mutations observed in 45–87% of infants after SD-NVP [12], [34], [13]. In our study, a significantly higher prevalence of NVP-R mutations was found by standard population sequencing among infants receiving SWEN (92%) and diagnosed with HIV infection by six weeks of age than those receiving only SD-NVP (38%). The prevalence of NVP-R remained higher with the SWEN regimen with low frequency clonal analysis, but because of small sample sizes we were not powered to detect a statistical difference. Others have reported higher prevalence of NVP-R mutations among Ugandan infants receiving extended-dose NVP while enrolled in the SWEN trial and diagnosed with subtype A or D infection in the first six weeks of life than for infants receiving SD-NVP [20]. In our analysis, daily dosing of NVP did not alter the dominant NVP-resistant mutation selected in infants infected in the first six of weeks of life. The Y181C variant remained dominant as observed in studies of NVP-R following single-dose exposure in infants [12], [13] and in earlier studies of daily NVP monotherapy for treatment of infected individuals [35], [36].

Among infants born to SD-NVP exposed women and who escape *in-utero* or peripartum/early-breastfeeding, the potential for acquiring NVP-resistant HIV through breastfeeding continues, as NVP-R mutations are present in breast-milk in up to 65% of women exposed to SD-NVP [37]. In our study, NVP-R did not differ by receipt of SD-NVP or SWEN among infants infected through late-breastfeeding. In both groups, 15% of infants had NVP-R detectable at population levels, with 31% of SWEN and 40% of SD-NVP exposed infants acquiring NVP-R when low frequency variants were considered. The predominant transmitted variants, here, were Y188C/H and K103N, reflective of the most common mutations found in women following SD-NVP exposure [12]. Maternal HAART exposure also did not play a role in the resistance outcomes we observed in these infants, as genotyping was done prior to initiation of maternal HAART and therefore, not due to exposure to suboptimal NVP concentrations in breast-milk. Furthermore, in the few samples available for analysis from women who transmitted HIV through breast-milk, the mutations present in plasma by standard population sequencing or clonal analysis often differed from those detected in their respective infants, highlighting the potential compartmentalization of HIV variants within the breast and the complexity of breast-milk transmission [38], [37].

While it is well established that the use of single antiretroviral agents in HIV-infected individuals virtually guarantees selection of drug resistant virus, the efficacy of administering HAART to HIV-infected women for prevention of breast-milk transmission is currently under study [39]. However, preliminary data from one study suggest that even with the administration of maternal HAART, the risk of transmission of drug-resistant HIV through breastfeeding remains. In the Kisumu Breastfeeding Study (KiBS) in Kenya where NVP or protease-inhibitor based HAART was administered to women from 34 weeks gestation through 6 months postpartum, 67% of 24 infants had either lamivudine and/or NVP-resistant HIV [40]. Since continued breastfeeding improves child survival, even for HIV-infected infants [41], use of antiretroviral drugs in the mother and or infant to prevent breast-milk transmission is currently the only available option for preventing HIV infection to the infant in resource-constrained settings, but, as seen here, this practice carries an inherent risk of drug resistance.

NVP-R as a consequence of prophylaxis to prevent MTCT is highly relevant because treatment in resource-constrained settings relies on NVP as a key component of HAART. NVP-R variants arising after SD-NVP are eventually replaced in blood by wild-type virus [12], [13], and so NVP-based HAART remains the first-line treatment regimen. In the context of extended NVP prophylaxis, a higher proportion of SWEN-exposed Ugandan infants had detectable NVP-R at population levels at six months of age than did SD-NVP exposed infants (100% of 7 vs. 17% of 6) [20]. However, in our study, NVP-R variants decayed in 70% of infants exposed to SWEN or SD-NVP by a median of 12 months of age, but remained at low frequencies in 86% of infants. The clinical implications for infants of this NVP-R and its persistence at low frequencies, however, are unclear. One small study showed a higher risk of virologic failure for SD-NVP-exposed than for unexposed infants on NVP-based HAART initiated at a median of 8 months [19]. Time to treatment initiation following prophylaxis in infants may also be critical, as SD-NVP exposed women who initiated NVP-based HAART less than six months after delivery had a higher risk of virologic failure than those who started treatment after six months [19]. Recent evidence showed that early therapy in HIV-infected infants can improve survival [42], which has led the WHO to recommend early HAART for all HIV-infected infants less than 12 months of age [43]. Therefore, if the findings on persistence of NVP-R from the Indian and Ugandan SWEN trials are confirmed in a large number of subtype A, C, or D HIV-infected infants, then this will influence the use of NVP as part of first line therapy, if extended NVP prophylaxis is implemented.

Our study has some limitations. First, unlike other studies of SD-NVP prophylaxis, we were unable to test for NVP-R at a fixed time point, although the median age at genotyping for infants infected *in utero* or through peripartum/early-breastfeeding was similar to other published studies. Assessing NVP-R at low frequencies using clonal analysis is limited by the number of clones required to detect NVP-R variants and may underestimate resistance at frequencies <5%. However, cloning did allow comprehensive assessment at all eight known sites of NVP-R in HIV reverse transcriptase. In addition, due to the limited number of maternal samples available for genotyping at six months and lack of breast-milk samples, drug resistance mutations present in plasma and breast-milk could not be determined or correlated with HIV transmission.

In summary, our results suggest the extended-dose NVP regimen used in the SWEN trial to prevent breast-milk transmission of HIV does carry a higher risk of infection with drug resistant virus than does SD-NVP in subtype C-infected infants, and that the risk of infection with NVP-R variants at low frequencies continues through the first year of life with breastfeeding. Given the high mortality associated with pediatric HIV infection in the first year of life, the complexities of early infant diagnosis, and limited resources for initiating HAART in infants in settings where breastfeeding itself is critical for survival, the primary goal should be to prevent peripartum and breast-milk transmission with infant antiretroviral prophylaxis. As other prevention options become available, this increased risk could be revisited.
